# COGNITIVE GROWTH AND DISPERSION AT MIDLIFE: IMPLICATIONS FOR COGNITIVE MAINTENANCE AND RESERVE

**DOI:** 10.1093/geroni/igae098.2650

**Published:** 2024-12-31

**Authors:** Chandra Reynolds, Anqing Zheng, Elizabeth Muñoz, Robin Corley, Sally Wadsworth

**Affiliations:** University of Colorado Boulder, Boulder, Colorado, United States; University of Colorado Boulder, Boulder, Colorado, United States; The University of Texas Austin, Austin, Texas, United States; University of Colorado Boulder, Boulder, Colorado, United States; University of Colorado Boulder, Boulder, Colorado, United States

## Abstract

Dispersion in performance across cognitive tasks provides a unique perspective of cognitive functioning beyond overall performance and may be predictive of poorer memory and dementia risk in older adults. What dispersion indexes at earlier age periods is unclear, although a lifespan perspective may provide an earlier window on cognitive specialization and reserve at the cusp of midlife. We sought to evaluate individual differences in dispersion in growth from adolescence and at midlife. Using twin and sibling data from the Colorado Adoption Twin Study of Lifespan behavioral development and cognitive aging (CATSLife1; N = 1245, MAge = 33.30, SD = 5.00, 53% Female), we evaluated dispersion using a latent model approach across tasks from a specific cognitive ability (SCA) battery, referencing age 16 and CATSLife1 assessment norms, and WAIS-III subtests at CATSLife1. At midlife, overall performance was modestly positively correlated with dispersion for the SCA tasks and the WAIS-III subtests (r=0.07 and 0.37). Referencing age 16 norms, SCA performance was correlated with dispersion at 0.88, suggesting differential growth from age 16 correlates strongly with CATSLife1 performance. Education predicted more dispersion referencing age 16 norms (B=0.41; CI=0.31 to 0.48], but not midlife dispersion for SCA or WAIS-III. Sibling similarity for the latent dispersion measures were moderate for identical (MZ) twins (r’s=0.55-0.86) and smaller for fraternal twins (DZ) (r’s=0.26-0.60), other biological siblings (r’s=0.10-0.36), and siblings in adoptive families (r’s=-0.15-0.11). Dispersion in growth may reflect differential specialization with implications to reserve, whereas variability in performance at midlife may be reflective of differences in maintenance.

